# Choice of antibody is critical for specific and sensitive detection of androgen receptor splice variant-7 in circulating tumor cells

**DOI:** 10.1038/s41598-022-20079-w

**Published:** 2022-09-28

**Authors:** Tanzila Khan, John G. Lock, Yafeng Ma, David G. Harman, Paul de Souza, Wei Chua, Bavanthi Balakrishnar, Kieran F. Scott, Therese M. Becker

**Affiliations:** 1 School of Medicine, Western Sydney University, Campbelltown, NSW 2560 Australia; 2 Medical Oncology, Ingham Institute of Applied Medical Research, Liverpool, NSW 2170 Australia; 3 Centre of Circulating Tumour Cells Diagnostics & Research, Ingham Institute for Applied Medical Research, Liverpool, NSW 2170 Australia; 4 School of Medical Sciences, University of New South Wales, Sydney, Australia; 5 South Western Sydney Clinical School, University of New South Wales, Liverpool Hospital, Liverpool, NSW 2170 Australia; 6 School of Science, Western Sydney University, Campbelltown, NSW 2560 Australia; 7 Department of Medical Oncology, Liverpool Hospital, Liverpool, NSW 2170 Australia

**Keywords:** Biological techniques, Cancer, Biomarkers, Medical research

## Abstract

Androgen receptor variant 7 (AR-V7) is an important biomarker to guide treatment options for castration-resistant prostate cancer (CRPC) patients. Its detectability in circulating tumour cells (CTCs) opens non-invasive diagnostic avenues. While detectable at the transcript level, AR-V7 protein detection in CTCs may add additional information and clinical relevance. The aim of this study was to compare commercially available anti-AR-V7 antibodies and establish reliable AR-V7 immunocytostaining applicable to CTCs from prostate cancer (PCa) patients. We compared seven AR-V7 antibodies by western blotting and immmunocytostaining using a set of PCa cell lines with known AR/AR-V7 status. The emerging best antibody was validated for detection of CRPC patient CTCs enriched by negative depletion of leucocytes. The anti-AR-V7 antibody, clone E308L emerged as the best antibody in regard to signal to noise ratio with a specific nuclear signal. Moreover, this antibody detects CRPC CTCs more efficiently compared to an antibody previously shown to detect AR-V7 CTCs. We have determined the best antibody for AR-V7 detection of CTCs, which will open future studies to correlate AR-V7 subcellular localization and potential co-localization with other proteins and cellular structures to patient outcomes.

## Introduction

Aberrant activity of the androgen receptor (AR) is central to prostate cancer development and first line therapy for metastatic prostate cancer is androgen deprivation therapy (ADT) which targets AR signalling^1,2^. However, ADT resistance inevitably occurs, and disease is then referred to as castrate resistant prostate cancer (CRPC).

The expression of altered AR proteins translated from alternative *AR* splice variants has been proposed as a mechanism of ADT resistance^3,4^. Expression of the AR splice variant 7 (AR-V7), is correlated with CRPC and is the most frequently identified disease associated variant. AR-V7 is proposed to be ligand independent and constitutively active as a nuclear transcription factor^5,6^.

**Figure 1:**
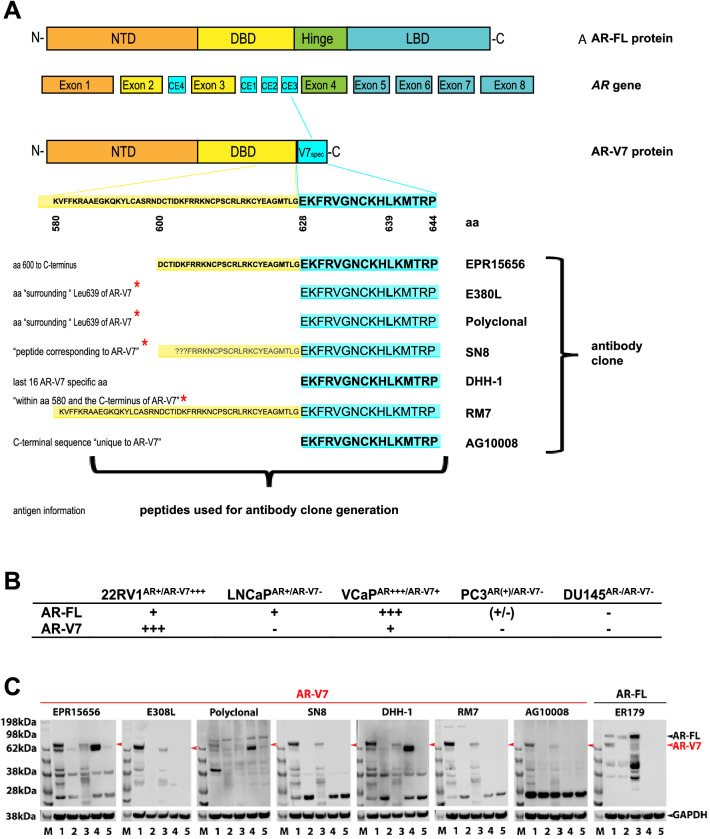
AR-V7 specific peptide and antigens for antibody generation. (**A**) Schematic presentation of the *AR*-gene encoding full length androgen receptor (AR-FL) and androgen receptor variant 7 (AR-V7) proteins. Amino acid (aa) sequences of cryptic exon (CE) 3 encoded AR-V7 specific domain (V7spec) and a section of DNA binding domain (DBD) shared with AR-FL are displayed and aa sequences representing antigens for antibody generation is indicated for the clones. Antigen information: as published by supplier or provided on request (*: information is considered ambivalent for four peptides, consequently shown aa sequences may not reflect exact antigen peptides, but are based on “informed assumption” that the V7 specific unique 16 aa are part of all peptides and uncertain proportions of the DBD shared with AR-FL as indicated); ???: DBD aa sequences uncertain; NTD: N-terminal domain; LBD: ligand binding domain; N-: N-terminal; -C: C-terminal. (**B**) Validation of AR-FL and AR-V7 mRNA expression in the indicated cell lines by ddPCR. – no detection; (+/−) low, but detectable; + detection; +++ high levels of AR-V7 or AR-FL copies; (**C**) Immunoblotting of total protein lysates from the indicated cell lines for AR-V7 (separate gels for each antibody, left), or AR-FL (right) using the indicated antibodies in reference to GAPDH (cropped from probing of the same membrane shown below each plot). M: size marker, 1: 22RV1^AR+/AR−V7+++^, 2: LNCaP^AR+/AR−V7−^, 3: VCaP^AR+++/AR−V7+^, 4: PC3^AR(+/−)/AR−V7−^, 5: DU145^AR−/AR−V7−^.

Splicing of the *AR* gene including exon 1, 2 and 3 together with a cryptic exon 3E (CE3) results in the *AR-V7* transcript (Fig. 1A). The unique cryptic exon has allowed the generation of highly sensitive and specific assays to detect AR-V7 at the mRNA level^7–10^. Importantly, given the general lack of matching tumor tissue for biomarker analysis at the CRPC stage these methods have been used to successfully detect AR-V7 transcripts from liquid biopsies, such as urine, plasma, exosomes and circulating tumor cells (CTCs) with the most reliable data originating from AR-V7 analysis in CTCs^11^. Our recent metanalysis emphasises the potential of AR-V7 detection in liquid biopsies as clinical biomarker, as it demonstrates significant correlation with patient survival overall and in context of specific treatment^1^. The presence of full-length *AR* (AR-FL) and *AR-V7* in CTCs has been investigated at the RNA level in a number of studies and CTC-based *AR-V7* was found to correlate with metastatic CRPC and primary resistance to abiraterone and enzalutamide^8,12–15^.

The AR-V7 protein has 16 distinctive C-terminal amino acids, encoded by an alternate cryptic exon 3 producing a unique AR-V7 C-terminal protein domain, allowing for generation of specific antibodies. To our knowledge seven antibodies are now commercially available designated to specifically detect the AR-V7 protein and have been raised to C-terminal peptides (Fig. 1A). AR-V7 protein detection opens opportunities for immunohistological analysis, however as outlined above tissue is rarely available for advanced prostate cancer analysis. Again, liquid biopsy derived CTCs lend themselves for immunocytostaining of AR-V7 to add to mRNA information that is readily obtained from these samples. Indeed, Scher et al. reported that information regarding AR-V7 subcellular localization within CTCs may add important information correlating to disease progression and therapy response^13,16,17^. This is an important finding as it potentially increases the value of AR-V7 screening as a biomarker in prostate cancer. Additionally, cellular AR-V7 protein analysis may enable future detailed investigations into interactions of AR-V7 with other proteins and nucleic acids to help understanding its CRPC related functions.

Here, using a cohort of well characterised prostate cancer cell lines with known and experimentally validated AR-V7 expression, we thoroughly tested the seven commercially available AR-V7 antibodies for their ability to truly detect AR-V7 by immunoblotting and immunocytostaining. Our findings highlight sensitivity, specificity and cross reactivities of antibodies and point towards an antibody of choice for AR-V7 immunocytostaining of CTCs. The antibody prioritised in this study performed well when employed for detection of CTCs from CRPC patients by immunocytostaining. Our finding is highly relevant to enhance AR-V7 utility to screen patients for therapy decisions or to stratify patients for relevant clinical trials.

## Methods

### Cell lines

22RV1, LNCaP, VCaP, PC3 and DU145 prostate cancer cell lines are here referred to as 22RV1^AR+/AR−V7+++^, LNCaP^AR+/AR−V7−^, VCaP^AR+++/AR−V7+^, PC3^AR(+/−)/AR−V7−^, DU145^AR−/AR−V7−^ according to their published and in this study validated AR-FL and AR-V7 expression^18–20^ and cells were grown in DMEM supplemented with 10% Foetal Bovine Serum (FBS), 2 mM l-Glutamine, 4 nM HEPES or MEM supplemented with 10% FBS, 2 mM l-Glutamine, 4 nM HEPES at 37 °C in 5% CO_2_. Cell lines were obtained directly or through Australian distributers (Merck/Sigma-Aldrich, Castle Hill; Invitro, Lane Cove, Australia) of the European Collection of Authenticated Cell Cultures (ECACC) or American Type Culture Collection (ATCC) and tested to be mycoplasma free (MycoAlert Mycoplasma Detection Kit, Lonza, Rockland, USA) and STR authenticated (AGRF, Melbourne, Australia). Cells were seeded at approximately 30–40% confluency and harvested after 72-h culture for immunoblotting and gene expression analysis.

### Antibodies

Six rabbit anti-human-AR-V7 antibodies were compared in this study: clone EPR15656 (Abcam, VIC, Australia), clone E308L and “polyclonal antibody” (Cell Signalling, Danvers, MA, USA), clone SN8 (Creative Diagnostic, Shirley, NY, USA), clone DHH-1 (RQ4683, Assay Matrix, VIC, Australia), and clone RM7 (RevMab Biosciences, San Francisco, CA, USA), as well as the mouse anti-human-AR-V7 clone AG10008 (Precision Antibody, Columbia, MD, USA). The available information of antigens used for the anti-AR-V7 antibody generation is shown in Fig. 1A. Additional antibodies used in this study are: mouse anti-human AR-FL, clone ER179 (Abcam, NSW, Australia), rabbit anti-GAPDH clone 14C10 (Cell Signaling, VIC, Australia), Alexa fluor 488 goat anti-rabbit IgG (H + L) (LOT 1423009) or Alexa fluor 488 goat anti-Mouse (H + L) (LOT 1252783) (Life technologies, Eugene, OR, USA), horseradish peroxidase-labelled donkey anti-Rabbit IgG (1:1000 dilution) (Lot 9526417, GE Healthcare, Buckinghamshire, UK) or sheep anti-mouse IgG, Horseradish Peroxidase linked F(ab^’^)_2_ fragment (1:1000 dilution) (Lot 312511, Amersham, GE Healthcare, Buckinghamshire, UK) and Alexa fluor 555 Phalloidin (Abcam, NSW, Australia).

### Droplet digital PCR (ddPCR)

In brief, total RNA was extracted with ISOLATE II RNA Mini Kit (Bioline, London, UK) from approximately 5 × 10^6^ cells. Quality and quantity of RNA was tested using a fragment analyser (5200 Fragment Analyzer System, CA, USA). cDNA was synthesised from 1 µg of total RNA per cell line using the SensiFAST cDNA Synthesis Kit (Bioline, London, UK). ddPCR to detect AR-V7 and full-length AR (AR-FL) was performed as described previously^15^. Quality of RNA was confirmed by conducting GAPDH ddPCR as described previously^11^.

### Western blotting

Approximately 1 × 10^6^ cultured cells were harvested and lysed in RIPA lysis buffer (50 mM Tris–Cl pH7.5, 150 mM NaCl, 0.5% Triton X-100, 2 mM EDTA, 2 mM EGTA 25 mM NaF, 10% glycerol) containing 1 × protease inhibiters (Roche, Basel, Switzerland) for 30 min placed on ice, followed by maximum microfuge centrifugation speed (11,700×g, 4 °C, 20 min) and recovery of supernatant. Protein concentrations were determined using the DC protein assay kit (Bio-Rad Laboratories, Hercules, CA). 30 µg total protein per sample was separated on 4–12% Bis–Tris gels (Invitrogen, Life Technologies) and transferred to Polyvinyl difluoride (PVDF) membrane (Amersham, GE Healthcare, Buckinghamshire, UK). Membranes were incubated with primary antibodies (dilutions see Supp. Table S1) overnight under gentle agitation at 4 °C. After three Tris-buffered saline with 0.1% Tween 20 detergent (TBS-T) washes, membranes were incubated with horseradish peroxidase-conjugated donkey anti-rabbit IgG (1:1000 dilution) or sheep anti-mouse IgG, horseradish peroxidase linked F(ab^’^)_2_ fragment (1:1000 dilution) for 1 h at room temperature and again washed three times. Membranes were developed using Western Lightning ™ Plus-ECL Enhanced Luminol Reagent Plus (LOT 275–13,481) and Western Lightning ™ Plus-ECL Oxidizing Reagent Plus (LOT 265-13481) (PerkinElmer, Waltham, MA, USA) and imaging was performed with an Odyssey imager (LI-Cor Biosciences, Lincoln, NE).

### Immunocytostaining of cell lines and PBMC

For each cell line approximately 20,000 cells were seeded on sterile, round coverslips in 12-well plates and cultured for 72 h followed by fixation with 3.7% paraformaldehyde for 10 min. In case of PBMC probing cells were cytospun onto superfrost + slides. Cells were permeabilized with 0.2% Triton-X for 10 min and blocked using 10% goat serum in PBS for 30 min. Primary antibodies were diluted in 0.5% FBS in PBS (Supplementary Table S1) and incubated for 1 h. Secondary antibodies conjugated with Alexa fluor 488 goat anti-rabbit IgG (H + L) (1:5000) or Alexa fluor 488 goat anti-mouse (H + L) (1:5000) were diluted in PBS with 0.5% goat serum and incubated 30 min. Cells were stained with Alexa fluor 555 phalloidin for 0.5 h followed by nuclear staining by using 1 × Hoechst (Fluxion, San Francisco, CA, USA) in PBS for 10 min. Coverslips were mounted with Pro Long ™ Glass Antifade Mountant (Eugene, OR, USA). Monochrome images were taken with Olympus IX71 microscope (Olympus, Tokyo, Japan) at × 20 magnification with identical acquisition settings, below any pixel intensity saturation in the brightest cell labelling conditions.

### CTC enrichment and immuncytostaining

To validate AR-V7 CTC detection six prostate cancer patients with advanced CRPC, likely to yield high CTC counts, were recruited. For each patient, 2 × 9 mL peripheral blood was collected into 2 EDTA vacutubes (Greiner Bio-One) and processed within 24 h. 2 × 9 mL blood was used to isolate CTCs using RosetteSep™ CTC enrichment cocktail containing anti-CD36 (Stemcell Technologies, Victoria, Australia) according to supplier’s instructions. In brief, blood was incubated with antibody cocktail for 10 min and then diluted with 2% FBS in PBS as recommended by manufacturer, transferred to a Sepmate tube containing lymphoprep density gradient medium (Stemcell technologies, VIC, Australia) and centrifuged at 1200xg for 10 min. The supernatant with cellular layer was recovered and topped up to 50 mL with 2% FBS in PBS and gently mixed. After a 10-min 300xg spin, the supernatant was discarded, and cells were suspended in residual fluid by gentle tapping. Cells were washed once with PBS and spun again (300xg, 10 min), resuspended in 1.5 mL PBS and transferred to a well of a 24-well glass bottom plate (Greiner Bio-One GmbH, Frickenhausen, Germany) coated with 3.5ug of CellTak (FAL354240, InVitro technologies, VIC, Australia) per cm^2^. After spinning the cells onto the glass (200 × g, 10 min) immunocytostaining was essentially performed as above including anti-AR-V7 (E308L or EPR15656) staining and probing for CD45 to exclude lymphocytes and Hoechst to secure nucleated cellular identity. All patient CTC samples were stained in parallel to a positive control 22RV1 sample. To identify CTCs the definition CD45-, AR-V7 + and Hoechst positive was used, where AR-V7 positivity was determined by the parallel 22RV1 probing. Typically, weak but clear AR-V7 staining in 22RV1 was around an intensity value of 2000 (AR488 channel, Olympus cellSens Dimension image analysis software). Consequently, events with intensities below 2000 were considered AR-V7 negative (not CTCs) for patient CTC samples. Images in Fig. 4 are displayed using pseudocolors to allow for merging of images from various channels.

### Image analysis and statistics

Image J (1.53c, National Institute of Health, USA) was used for RGB stacking and merging of immunostaining images from cell lines before doing quantitative image analysis using CellProfiler (Broad Institute, MIT, Massachusetts, USA) an automated image analysis software to measure biological phenotypes in images^21^. CellProfiler segmented cell data for at least 150 cells per sample (nucleus and cytoplasm) based on staining and extracted data on nucleus, cell body and cytoplasm and AR-V7 intensity were saved in excel to transfer to Konstanz Information Miner (Knime)^22^. The quantitative data from CellProfiler was used in Knime to compare the intensity of AR-V7 detected by different antibodies as well as cellular localization of AR-V7.

### Ethical approval and consent to participate

All methods were performed in accordance with the relevant guidelines and regulations and the study was approved by the South Western Sydney Local Health District Human Ethics Committee (HREC/13/LPOOL/158). Written informed consent was obtained from all patients participating in the study.

### Consent for publication

All authors agree on the submitted version of the manuscript.

## Results

For any antibody to be selective for AR-V7, it must recognise a C-terminal peptide epitope corresponding to the 16 amino acid peptide sequence (EKFRVGNCKHLKMTRP) unique to AR-V7, encoded by the cryptic exon 3. Supplier information regarding the exact antigens used for antibody generation is considered imprecise for five of the seven antibodies tested here. Nevertheless, one can deduce that the entire 16 amino acids or most are part of any antigenic peptide used for antibody generation. Three of the antibodies are known or implied to have antigen peptides additionally containing parts of at least the DNA binding domain (DBD) shared by AR-V7 and AR-FL (Fig. 1A).

To be able to thoroughly compare these antibodies, AR-V7 status of several prostate cancer cell lines was validated. ddPCR confirmed high and detectable AR-V7 in 22RV1 ^AR+/AR−V7+++^ and lower but readily detectable transcript AR-V7 expression in VCaP^AR+++/AR−V7+^, while AR-V7 is negative for LNCap^AR+/AR−V7−^, PC3^AR(+/−)/AR−V7−^, DU145^AR−/ARV7−^, ddPCR also confirmed known AR-FL status for all lines (Fig. 1B)^15^.

To test whether all of the anti-AR-V7 antibodies interact with a protein of the expected AR-V7 size of ~ 80 kDa in our AR-V7 expressing cell lines, or whether the antibodies may cross react with other proteins we first tested the antibodies by immunoblotting of full protein lysates from all cell lines (Fig. 1C). We also included an anti-AR-FL antibody to clarify whether the AR-V7 antibodies identified protein bands of AR-FL size. None of the specific anti-AR-V7 antibodies produced a band considered AR-FL. Interestingly, only the anti-AR-V7 antibody clones E308L, SN8, RM7 and AG1008 produced a distinct band appearing around the expected AR-V7 size for AR-V7 positive 22RV1^AR+/AR−V7+++^ and VCaP ^AR+++/AR−V7+^ cells, while not detecting anything above background in AR-V7 negative cell lines in that protein size range. However, there was clearly some cross-reactivity detected for proteins of smaller size. We considered E308L was the “cleanest” antibody with negligible cross-reactivity detected for AR-V7 negative cell lines. SN8, RM7 and especially AG1008 produced strong reaction to proteins of sizes other than 80 kDa in all, including AR-V7 negative cell lines with one band appearing relatively dominant just below the 28 kDa range.

Two anti-AR-V7 antibodies (EPR15656 and DHH-1), while detecting protein bands corresponding to AR-V7 size in 22RV1^AR+/AR−V7+++^ and VCaP ^AR+++/AR−V7+^ cells, additionally detected a very strong band in AR-V7 negative PC3^AR(+/−)/AR−V7−^ cells, while additional bands across cell lines evidenced further cross-reactivity for these antibodies. The prominent PC3^AR(+/−)/AR−V7−^ protein band was just below the expected AR-V7 size. AR-V7 detection with the polyclonal antibody proved to be highly nonspecific. (Fig. 1C). Complete raw data (images of all immunoblots) are provided as Supplementary Fig. S1.

Although deemed very unlikely that PC3^AR(+/−)/AR−V7−^ with undetectable AR-V7 transcript express a slightly truncated form of AR-V7, we wished to rule out AR-V7 identity of this band detected close to 80 kDa. Firstly, we conducted protein Blast searches (blast.ncbi.nlm.nih.gov/Blast.cgi?PAGE = Proteins) of the full AR-V7 specific 16 amino acid sequence as well as the sequence from amino acid 580 and 600 (see Fig. 1A) to the C-terminus of AR-V7 against the human protein database (https://www.uniprot.org), which identified only the AR-V7 splice variant and for the 580/600 to C-terminus peptide additionally to AR-V7 the partially homologue AR variant 5 (see ref^23^ for review of AR variants). Additionally, we were able to elute the ~ 62 kDa PC3^AR(+/−)/AR−V7−^ band of interest from a gel to perform mass spectroscopy and the retrieved data confirmed our Blast data with no proteins detected that share homology to AR-V7 or AR-FL in the excised protein band of interest from PC3^AR(+/−)/AR−V7−^ cells. Out of interest the same was done for the 28 kDa band as cross reactivities for that size were detected by several anti-AR-V7 antibodies, no AR(-V7) homology was found. The datasets generated for these analyses are available (https://doi.org/10.26183/7d43-ze13).

With Western analysis already pointing towards clear specificity differences between the tested antibodies we excluded two antibodies from further analysis, the polyclonal due to lack of specificity for AR-V7 detection sensitivity and specificity by Western analysis, and the mouse monoclonal AG10008. The latter was excluded due to very strong cross-reactivity in all cell lines with a protein band at ~ 28 kDa compared to specific AR-V7 band intensity and since ultimately, we aim to perform AR-V7 detection by immunocytostaining of CTCs. In our established CTC workflow antibodies of rabbit origin are more easily integrated for technical reasons. Initial immunocytostaining analysis of the five remaining antibodies was performed focusing on the AR-V7-positive cell line, 22RV1^AR+/AR−V7+++^ and the AR-V7-negative cell line LNCap^AR+/AR−V7−^.

**Figure 2:**
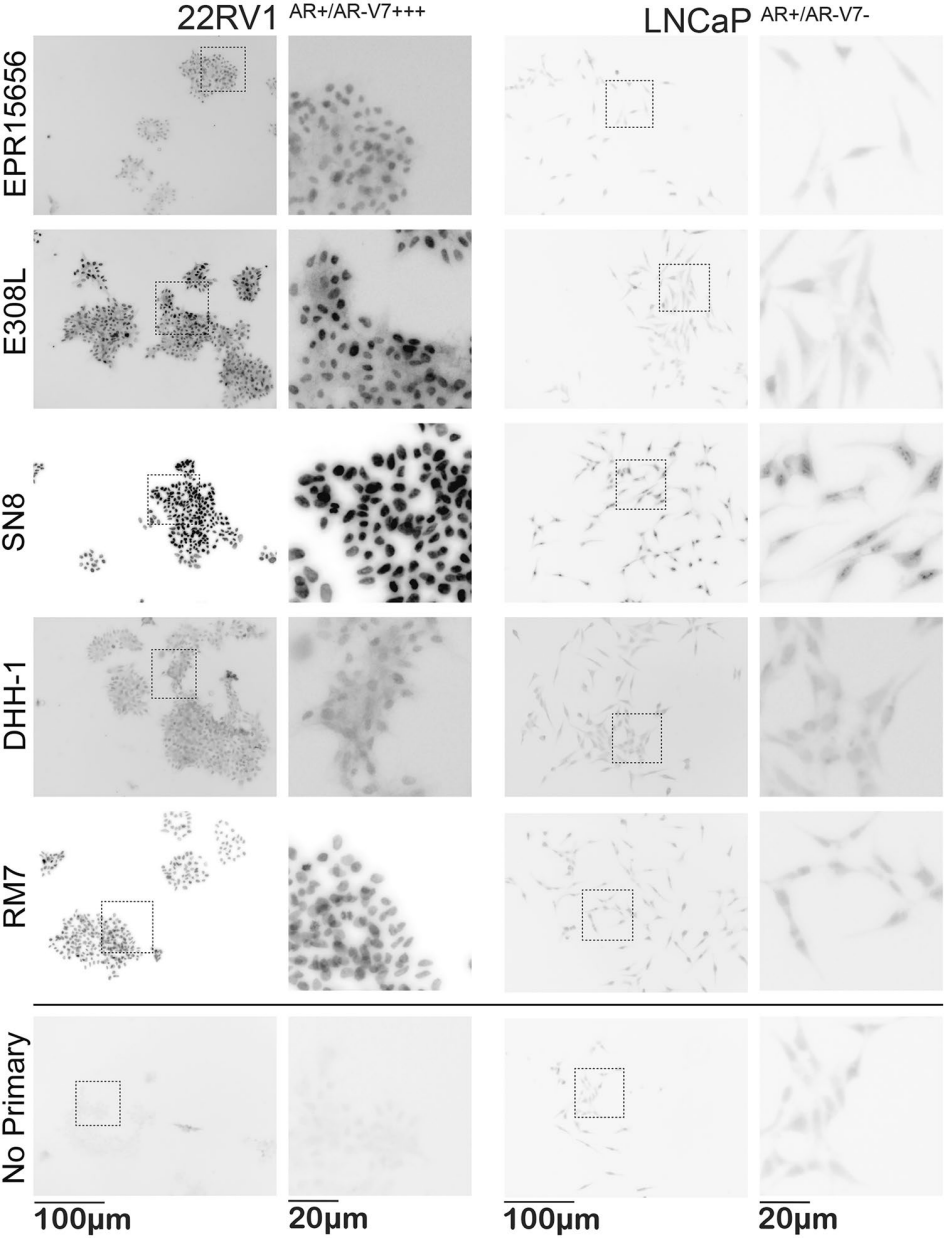
AR-V7 staining with different antibodies in AR-V7 positive and negative cells. Immunocytostaining was performed with the indicated antibodies on 22RV1^AR+/AR−V7+++^ and LNCaP^AR+/AR−V7−^ cells in comparison to no-primary antibody controls. Images were acquired with identical acquisition settings, with no pixel intensity saturation in the brightest cell labelling conditions. This enables quantitative comparison of intensity values across all antibodies and cell lines. Here, monochrome images are presented inverted, allowing easier visual detection of low intensity labelling patterns. Overview visual fields of stained cells are shown to the left with higher magnification images for representative regions (dotted boxes) to the right.

**Figure 3:**
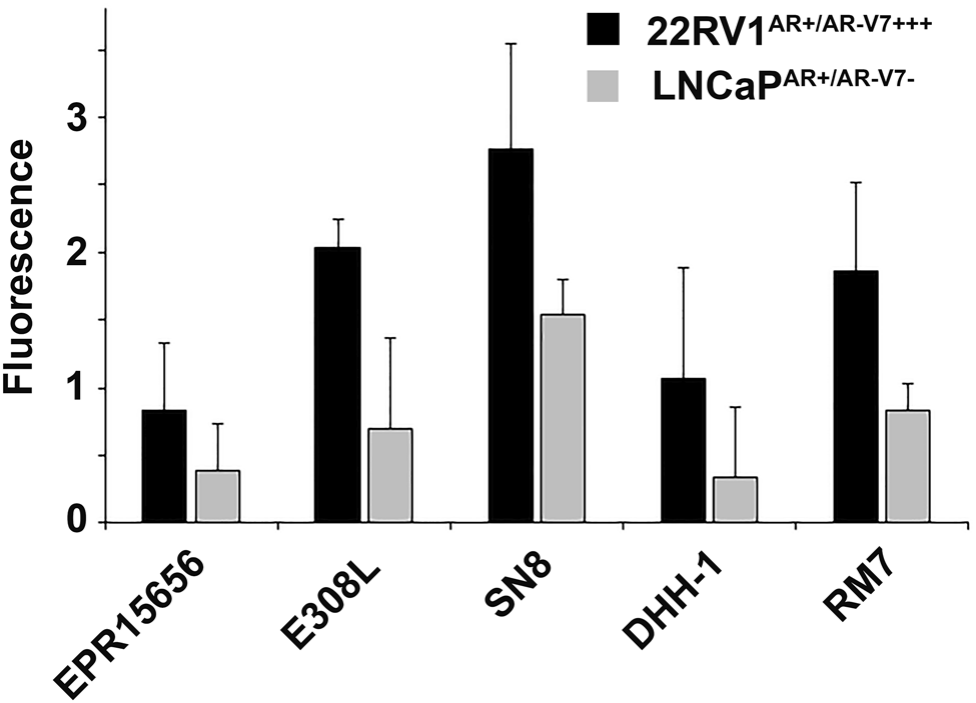
AR-V7 staining nuclear intensity. Cells (imaged as presented in Fig. 2) were segmented using CellProfiler based on identification of Hoechst as a nuclear marker and Alexa fluor 555 phalloidin as a cell body marker. This permitted selective measurement of AR-V7 labelling intensities in individual whole cells, as well as in nuclear and cytoplasmic compartments, per cell. Here, nuclear AR-V7 labelling intensity (average and standard deviation of at least 150 cells per condition) is depicted after normalisation to control values (no primary antibody labelling), allowing comparison of antibody signals in 22RV1^AR+/AR−V7+++^ and LNCaP^AR+/AR−V7−^ cells. Fluorescence (Y-axis): in arbitrary units.

Representative immunocytostaining images of all remaining antibodies in the two cell lines used for monochromatic analysis of staining intensity and subcellular localization are shown in Fig. 2. In comparison to “no primary” control staining we analysed intensity of staining, and subcellular localization of staining as specific AR-V7 staining is expected to be predominantly nuclear^24^. Initial subjective visual analysis clearly favours the E308L and SN8 antibodies that show distinct nuclear AR-V7 staining in 22RV1^AR+/AR−V7+++^, however SN8 produces what appears to be cross-reactivity with nucleolar structures in the negative control LNCap^AR+/AR−V7−^ cells. EPR15656, DHH-1 and RM7 appear to produce less distinct staining in 22RV1^AR+/AR−V7+++^ vs LNCap^AR+/AR−V7−^ cells. Analysing images using unbiased digital image analysis essentially confirmed these observations, presented in Fig. 3 for nuclear intensity. E308L produced the second highest nuclear staining intensity after SN8 in 22RV1^AR+/AR−V7+++^ cells, and that corresponded to the second lowest nuclear staining intensity in AR-V7 negative LNCap^AR+/AR−V7−^. Since the staining in LNCap^AR+/AR−V7−^ can be attributed to unspecific antibody binding, E308L is the antibody with the best signal detection to noise ratio for AR-V7 immunocytostaining.

**Figure 4:**
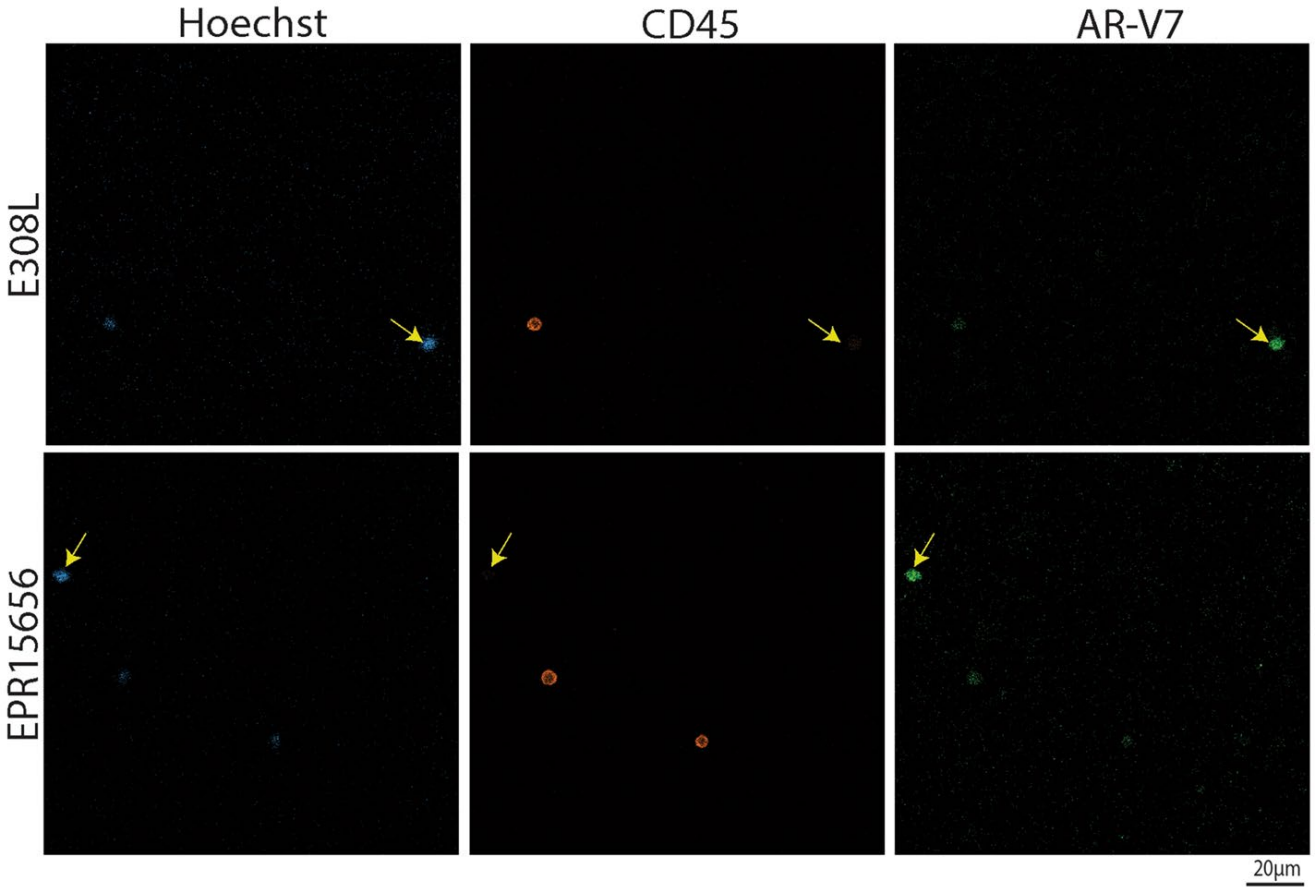
AR-V7 CTC detection. CTCs were identified based on AR-V7 staining. In brief, nucleated (blue, Hoechst) events were included in CTC counts if negative for CD45 (orange) and positive for AR-V7 (green) using the indicated anti-AR-V7 antibodies. AR-V7 was considered positive if intensities (Olympus cellSens Dimension image analysis software) were comparable or above intensities of cells weak but clearly identifiable (by operator) AR-V7 staining in 22RV1 (included as positive controls in each staining run).

The goal of the presented antibody comparison was to find the best suited anti-AR-V7 antibody to probe and analyse AR-V7 in CTCs. With E308L emerging as the favourite candidate anti-AR-V7 antibody while EPR15656 was previously published to be used for AR-V7 immunocytostaining in CTCs^20,24^, a final validation of both antibodies, E308L and EPR15656, was by detection of CTCs isolated from a small number of CRPC patients (to validate antibody staining this pilot study focussed on patients with advanced CRPC only, see Table 1). It is evident that E308L detected prostate cancer patient CTC numbers are consistently higher, indicating higher sensitivity of E308L CTC detection. Additionally, heterogeneity of AR-V7 expression is apparent as detection efficiencies are between 7–308% higher using E308L (Table 1). Representative CTC detection with both antibodies is shown in Fig. 4. Importantly, using both antibodies on three healthy donor PBMCs, confirmed negligible background staining for residual blood cells with the anti-AR-V7 antibodies in blood cells (Supplementary Fig. S2).

**Table 1:** AR-V7 staining CTC detection by antibody in advanced CRPC patients.

Patient	Age	Clinical notes	CTC counts	
			E308L	EPR15656
1	75	High grade disease, lymph node involvement (PSMA PET)	38	23
2	78	Widespread pelvic disease with vesical and rectal fistula	29	20
3	77	Widespread bone metastases, on chemotherapy, anemia	69	64
4	85	Widespread bone metastases, starting clinical trial (failed standard therapies)	45	5
5	84	Aggressive soft tissue disease in neck lymph nodes and meningeal metastases despite chemotherapy	173	95
6	77	Metastatic bone disease, Gleason grade 4 + 5 = 9	184	45

CTCs were enriched (RosetteSep) and immunocytostained for detection. Nucleated (Hoechst staining) events were included in CTC counts if negative for CD45 and positive for AR-V7 using the indicated anti-AR-V7 antibodies.

## Discussion

Here we compared various antibodies to establish specific detection of AR-V7 by immunocytostaining with ultimate focus to detect AR-V7 positive CTCs from prostate cancer patient bloods. Initially PCa cell lines with known and here confirmed status of AR-V7 expression (Fig. 1B) provided the tools to precisely judge cross-reactivity of antibodies in AR-V7 negative cells while specific staining and its subcellular localization could be determined in AR-V7 expressing cells. Western analysis helped to determine sensitivity and specificity of antibodies first, since higher cross-reactivity for immunocytostaining is likely mirrored by the appearance of cross-reactive bands in AR-V7 negative cells and for molecular weights other than the ~ 80 kDa of AR-V7 in positive cells. Indeed the “cleanest” antibody by Western analysis, E308L, in the end also emerged as our favoured antibody for immunocytostaining as well. The other antibody that performed highly for immunocytostaining in AR-V7 positive 22RV1^AR+/AR−V7+++^ cells, clone SN8, caused however clearly noticeable nuclear staining in AR-V7 negative LNCap^AR+/AR−V7−^ cells, interestingly of nucleolar appearance. This means use of this antibody for CTC detection likely would cause false positive AR-V7 detection in the cellular compartment that has been linked to AR-V7 activity^24^, affecting potential for “true” biomarker detection.

AR-V7 detection in CTCs by immunocytostaining has been reported previously using the ERP15656 antibody^20,24^. This prompted us to do a direct comparison of CTC detection in parallel patient blood samples with our favourite E308L as well as the ERP15656 antibody. For proof-of-concept, we focused on a small highly advanced CRPC patient cohort (Table 1) to increase likelihood of high CTC numbers. We also used unbiased CTC enrichment using lymphocyte depletions as AR-V7 has been reported more common in EpCAM-negative CTCs^25^. Interestingly the E308L did not only consistently detect more CTCs in the small cohort of 6 patients (Table 1), but did so marginally to dramatically, which likely reflects heterogeneous AR-V7 protein levels in patient CTCs. More work is needed to verify the extent and heterogeneity of AR-V7 levels in CTCs. So far EPR15656 staining has shown correlation of AR-V7 CTC staining with patient outcome^24^. Nevertheless, evaluation in larger patient cohorts is needed to clarify if AR-V7 detection in CTCs by immunocytostaining is better suited to predict patient outcome than detection by mRNA, or if indeed a combination of both methods may have benefit. A clear benefit of detecting the AR-V7 protein rather than only mRNA in CTCs is that it opens opportunities to evaluate cell by cell heterogeneity and how AR-V7 expression and sub-cellular localization is related to that of other proteins, which may not only add to our understanding of AR-V7 function but reveal ways of therapeutically targeting it in the future.

## Conclusion

Here we evaluated the commercially available antibodies against AR-V7 for utility in immunocytostaining of cell lines with known AR-V7 status and CRPC patient CTCs. The clone E308L emerged as the favoured antibody considering sensitivity and specificity as shown by immunoblotting and signal to noise ratio in immunocytostaining. With the growing attraction of liquid biopsies in diagnostic settings, identification of the best antibody to detect AR-V7 in CTCs may help to develop a standardised approach for AR-V7 screening in patient CTCs.

## Supplementary Information

Supplementary Figure 1.

Supplementary Figure 2.

Supplementary Information 1.

## Data Availability

All data generated in this study are included either in this article or in a supplementary table. Mass Spectroscopy datasets generated and/or analysed during the current study are available in the Western Sydney University ResearchDirect repository, https://doi.org/10.26183/7d43-ze13 .

## Abbreviations

aa: Amino acid
ADT: Androgen deprivation therapy
AR: Androgen receptor
AR-V7: Androgen receptor variant 7
CRPC: Castration-resistant prostate cancer
CTCs: Circulating tumour cells
CE3: Cryptic exon E3
DBD: DNA binding domain
ddPCR: Droplet digital PCR
FBS: Fetal Bovine Serum
AR-FL: Full length AR
Knime: Konstanz Information Miner
LBD: Ligand binding domain
NTD: N-terminal domain
PVDF: Polyvinyl difluoride
PCa: Prostate cancer
TBS-T: Tris-buffered saline with 0.1% Tween 20 detergent
