# Supplementary figures and images for: Choice of antibody is critical for specific and sensitive detection of androgen receptor splice variant-7 in circulating tumor cells
Source: Sci Rep. 2022 Sep 28;12:16159. doi: 10.1038/s41598-022-20079-w (PMC9519917; doi:10.1038/s41598-022-20079-w)

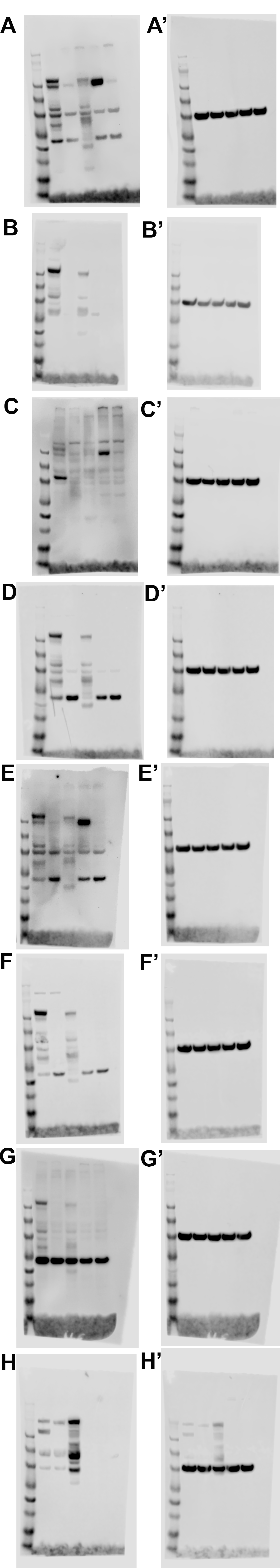

Supplement: Supplementary file 1 — Supplementary Figure 1. [file 41598_2022_20079_MOESM1_ESM.tif]

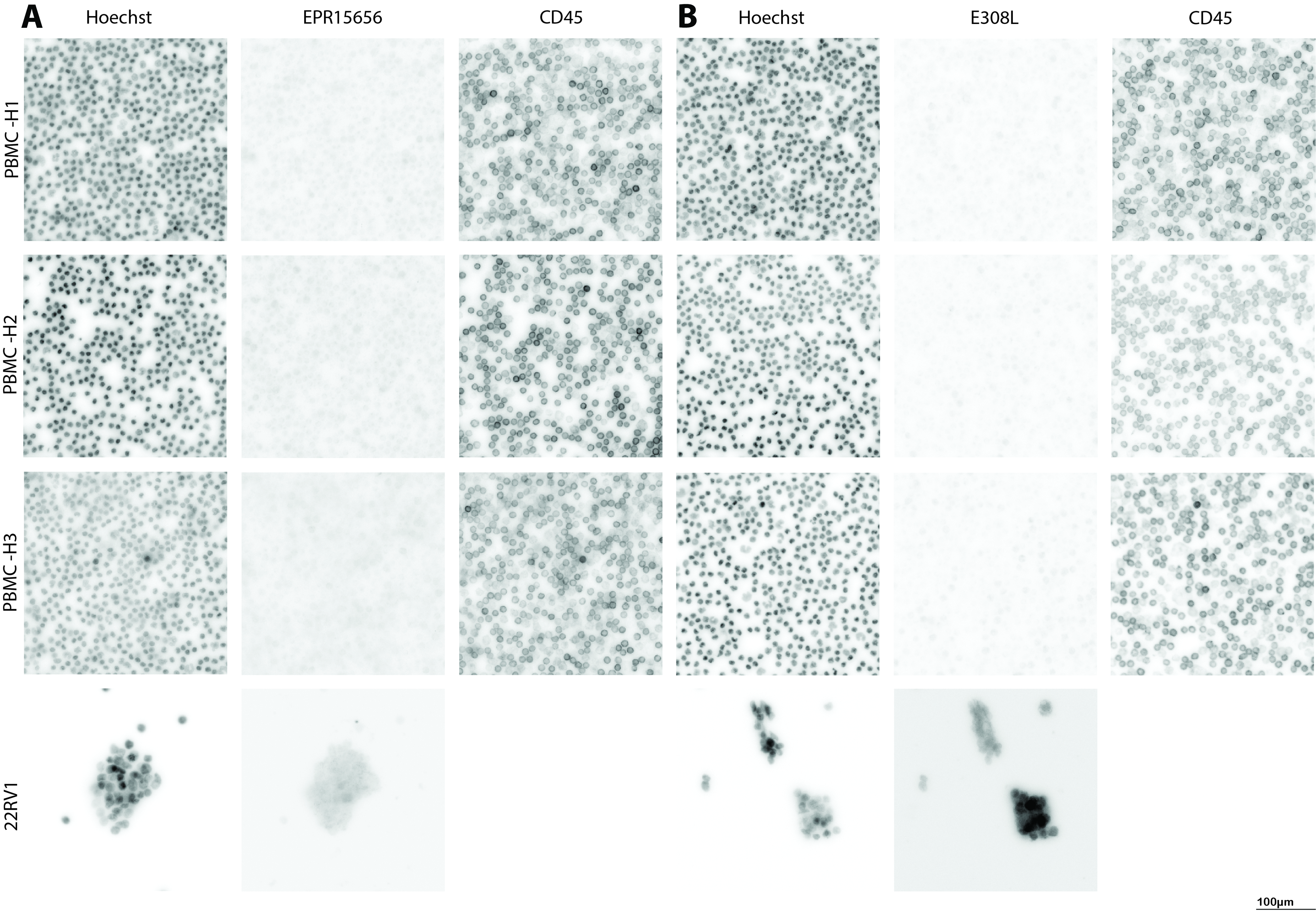

Supplement: Supplementary file 2 — Supplementary Figure 2. [file 41598_2022_20079_MOESM2_ESM.tif]
