# Supplementary material for: Choice of antibody is critical for specific and sensitive detection of androgen receptor splice variant-7 in circulating tumor cells
Source: Sci Rep. 2022 Sep 28;12:16159. doi: 10.1038/s41598-022-20079-w (PMC9519917; doi:10.1038/s41598-022-20079-w)
Supplement: Supplementary file 3 — Supplementary Information 1. [file 41598_2022_20079_MOESM3_ESM.docx]

**Supplementary Tables**

Supplementary Table S1 **Anti-AR-V7 antibodies and working dilutions**

| **Host [Clone]** | **Western**  **blot** | **Immuno-cytostaining** |
| --- | --- | --- |
| R [EPR15656] | 1:1000 | 1:100 |
| R [E308L] | 1:500 | 1:100 |
| R [polyclonal] | 1:1000 | 1:100 |
| R [SN8] | 1:2000 | 1:100 |
| R [DHH-1] | 1:500 | 1:50 |
| R [RM7] | 1:1000 | 1:100 |
| M [AG10008] | 1:250 | 1:100 |

Dilutions of antibodies. R: rabbit; M: mouse;

**Supplementary Figure Legends**

**Supplementary Figures 1**

Original uncropped images of unlabelled immunoblots for in-manuscript Figure 1C are presented depicting probing for: **A:** EPR15656, **A’:** A-re-probe for GAPDH (loading control); **B:** E308L, **B’:** B-re-probe for GAPDH (loading control); **C:** polyclonal, **C’:** C-re-probe for GAPDH (loading control); **D:** SN8, **D’:** D-re-probe for GAPDH (loading control); **E:** DHH-1, **E’:** E-re-probe for GAPDH (loading control); **F:** RM7, **F’:** F-re-probe for GAPDH (loading control); **G:** AG10008, **G’:** G-re-probe for GAPDH (loading control); **H:** ER179, **H’:** H-re-probe for GAPDH (loading control).

**Supplementary Figure 2**

Immunocytostaining was performed with the indicated antibodies on PBMCs isolated from three independent healthy donors, H1-H3, in comparison to 22RV1^AR+/AR-V7+++^ cells. Images were acquired with identical acquisition settings, with no pixel intensity saturation in the brightest cell labelling conditions. This enables quantitative comparison of intensity values across all antibodies and cells. Here, monochrome images are presented inverted, allowing easier visual detection of low intensity labelling patterns.
